# Perceptions of extended-release buprenorphine among people who received medication for opioid use disorder in jail: a qualitative study

**DOI:** 10.1186/s13722-024-00486-2

**Published:** 2024-09-12

**Authors:** Thomas J. Stopka, Rebecca Rottapel, Peter D. Friedmann, Ekaterina Pivovarova, Elizabeth A. Evans

**Affiliations:** 1 Department of Public Health and Community Medicine, Tufts University School of Medicine, 136 Harrison Avenue, Boston, MA 02111 USA; 2 Office of Research, University of MA Chan Medical School – Baystate and Baystate Health, Springfield, MA USA; 3 Department of Family Medicine and Community Health, University of Massachusetts Chan Medical School, 55 Lake Avenue North, Worcester, MA 01655 USA; 4 Department of Health Promotion and Policy, School of Public Health and Health Sciences, University of Massachusetts Amherst, 312 Arnold House, 715 North Pleasant Street, Amherst, MA 01003 USA

**Keywords:** Medication for opioid use disorder, Extended-release buprenorphine, Jails, Massachusetts, Qualitative

## Abstract

**Background:**

Incarceration provides an opportunity for health interventions, including opioid use disorder (OUD) treatment and prevention of opioid-related overdoses post-release. All FDA-approved forms of medication for OUD (MOUD) treatment were mandated in several Massachusetts jails in 2019, with some jails offering extended-release buprenorphine (XR-Bup). Little is known about patient perspectives on and experiences with XR-Bup in carceral settings.

**Methods:**

We conducted semi-structured interviews in 2022 with community-dwelling people who received MOUD during a recent incarceration in a Massachusetts jail. We asked participants about their experiences with and perspectives on XR-Bup while in jail. Qualitative data were double-coded deductively and reviewed inductively to identify emergent themes, which were structured using the Theoretical Framework of Acceptability (TFA).

**Results:**

Participants (n = 38) had a mean age of 41.5 years, were 86% male, 84% White, 24% Hispanic, and 95% continued to receive MOUD at the time of their interview, including 11% receiving XR-Bup. Participants who viewed XR-Bup favorably appreciated avoiding the taste of sublingual buprenorphine; avoiding procedural difficulties and indignities associated with daily dosing in carceral settings (e.g., mouth checks, stigmatizing treatment from correctional staff); avoiding daily reminders of their addiction; experiencing less withdrawal; having extra time for other activities, such as work; and reduction of diversion of MOUD within the jail setting. Participants who viewed XR-Bup less favorably preferred to maintain their daily dosing routine; liked daily time out of their housing unit; wanted to know what was “going into my body everyday”; and feared needles and adverse events. Participants also reported that jail clinicians used XR-Bup for patients who were previously caught diverting sublingual buprenorphine, suggesting limited patient participation in decision-making around XR-Bup initiation in some jails.

**Conclusion:**

People who received MOUD in Massachusetts jails had both favorable and unfavorable views and experiences with XR-Bup. Understanding these preferences can inform protocols in jails that are considering implementation of XR-Bup treatment.

**Supplementary Information:**

The online version contains supplementary material available at 10.1186/s13722-024-00486-2.

## Introduction

Opioid use disorder (OUD) and opioid-related overdoses associated with the current opioid crisis continue to present substantial public health and clinical challenges. In 2020, an estimated 2.7 million people in the US had OUD during the past 12 months [1]. Fatal overdoses reached nearly 112,000 for the 12-month period through June of 2023, according to federal estimates [2]. Fatal opioid-related overdoses increased five-fold over the past two decades in Massachusetts [3], with fentanyl implicated in more than nine of ten overdose deaths from 2019 to 2023.

People who use opioids (PWUO) are at elevated risk of incarceration [4] and, in the US, people with a history of incarceration have elevated overdose risks upon release from carceral settings [5, 6]. Correctional facilities present clinical and public health intervention touch points for the prevention and treatment of OUD and opioid-related overdoses [7].

Medications for opioid use disorder (MOUD), including opioid agonists (methadone), partial agonists (buprenorphine), and antagonists (extended-release naltrexone, XR-NTX), are effective in treating OUD [8–10], with buprenorphine and methadone associated with up to 59% reductions in fatal overdose risk [11]. To date, research on MOUD treatment among incarcerated populations, largely conducted outside the US, has recorded favorable outcomes [12, 13]. Despite MOUD treatment effectiveness and increased needs among people within corrections settings, MOUD treatment is not available in most correctional facilities across the US [14]. This gap in treatment access, however, has slowly begun to close in recent years, with potential opportunities to decrease OUD, overdose deaths [15, 16], and recidivism post-release [17].

The development of long-acting versions of MOUD (e.g., naltrexone and buprenorphine) offers great potential for use in correctional settings, where OUD rates are estimated to be approximately 25 times higher than among the general population, and medication diversion concerns abound [18]. However, low rates of community extended-release (XR) naltrexone continuation post-release, limited effectiveness in decreasing overdose risk [11], and modest opioid-use reductions in early XR-naltrexone trials have tempered enthusiasm [19].

In 2017, the FDA approved an XR-Buprenorphine (XR-Bup) formulation (Sublocade®; Indivior, Inc.) with subcutaneous injections of 100 mg or 300 mg buprenorphine delivered every four weeks. A weekly or monthly formulation (Brixadi™; Braeburn, Inc.) was FDA approved in 2023. Studies of Sublocade with community-based OUD patients have shown high retention and positive opioid-use, health-related, and community outcomes [21, 22]. While XR-Bup is expensive, cost analyses have indicated that XR-Bup is more affordable in correctional settings than other MOUD formulations in Australia [20]. Recent studies in the US indicate that XR-Bup in carceral settings are feasible and acceptable [21], and that patients on XR-Bup had fewer in-jail medical visits and increased community treatment retention following release when compared with standard daily sublingual buprenorphine (SLB) treatment [22], which can contribute to decreased opioid-related overdose risks.

Qualitative studies have begun to elucidate patient perspectives of XR-Bup following release from jails. In Sweden, Johnson and colleagues found that XR-Bup injections might have “social, practical, and psychological benefits compared to other formulations” but that such injections were “not perceived as an attractive option by all patients” [23]. In New York City jails, a small qualitative study (n = 16) found that XR-Bup “…satisfaction was attributed to reduced in-jail clinic and medication administration visits, perceived efficacy and blockade effects upon the use of heroin/fentanyl following release, and averting the risk of criminal activities to fund opioid use [24].” Barriers to XR-Bup retention included “post-injection withdrawal symptoms and cravings attributed to perceived suboptimal medication dosing, injection site pain, and lack of in-jail provider information about the medication” [24]. Little is known about patient experiences with and perceptions of XR-Bup in other carceral settings, nor the implications for treatment outcomes within such settings, and following release from jails.

During September of 2019, seven county jails and houses of correction (here forward “jails”) in Massachusetts initiated provision of all government-approved MOUDs following the passage of state legislation that mandated such treatment be made available [25]. In subsequent years, MOUD programming expanded and is now offered by most jails in Massachusetts. As MOUD treatment within carceral settings becomes more available in the US [26], a better understanding of the experiences with and outcomes related to different MOUD formulations is needed. Previous studies have focused on implementation experiences of jail-based MOUD treatment programs [27], barriers to MOUD treatment continuity post-release from jails in Massachusetts as described by jail staff, clinical providers [28] and community-based MOUD treatment providers [29]. XR-Bup treatment formulations have begun to be offered in Massachusetts jails, but experiences with and perspectives on XR-Bup among incarcerated people have been limited.

The Massachusetts Justice Community Opioid Innovation Network (JCOIN) is conducting an implementation study with treatment programs that began providing MOUD to people who are incarcerated in Massachusetts jails beginning in September 2019 [30]. Through the current study, which is a sub-analysis from the larger project, we aimed to assess XR-Bup perspectives and experiences among people who were recently released from local jails in Massachusetts. Our goal was to describe MOUD patient perspectives related to XR-Bup by highlighting contextual factors that could impact XR-Bup initiation and retention within jails and post-release to local communities.

## Methods

### Participants

Interviews were conducted with 38 individuals who received a Food and Drug Administration (FDA)-approved MOUD while incarcerated after Sept 1, 2019, in one of the eight county jails in Massachusetts that was offering MOUD by the time of recruitment. The counties were in Eastern and Western Massachusetts, in urban, suburban, and rural locations. Recruitment was conducted via flyers (n = 9) and word-of-mouth (n = 26). Flyers were posted or distributed in jail release packets and community locations where previously incarcerated individuals may gather (e.g., community opioid treatment programs (OTP), transitional housing). In addition, potential participants who previously gave permission to the jail to contact them following release were contacted directly and invited to participate. For three participants (n = 3), it was unknown how they learned about the study. Study details were explained to all potential participants and all who agreed to participate provided verbal consent. Interested individuals contacted research staff who provided study information and verified in-jail receipt of MOUD.

### Data collection

Individuals participated in 30-to-60-min semi-structured phone interviews from Fall 2021 through Summer 2022. Verbal consent was obtained by research staff prior to beginning the interviews. Participants were compensated $40. Interviews were digitally recorded, professionally transcribed, and redacted. Interviewers were female and male and included a social worker, anthropologist, epidemiologist, clinical psychologist, public health PhD candidate, and masters-level staff members; all had prior experience conducting qualitative research interviews. In addition, qualitative research experts on the team provided group and one-on-one training for the interviewers to ensure best practices and consistent approaches to data collection. The Baystate Health Institutional Review Board approved all study procedures.

### Data analysis

An initial codebook based on the interview guide questions was developed, with additional codes added as needed to reflect salient emerging topics in the data. Codes were refined using open coding and constant comparative methods, resulting in a codebook with 23 parent codes and 32 child codes. A coding team of four trained, master’s level staff members (SF, EB, PD, RS, see acknowledgements) deductively coded four initial transcripts concurrently and met to review coding application agreement and refine coding definitions until sufficient agreement was reached. Thereafter, the four staff worked in two dyads, coding each transcript independently and meeting with their dyad partner to resolve any discrepancies in coding. Both dyads (n = 4) met regularly during the coding process to discuss ongoing code applications and to reconcile any final code definitions and applications to ensure all were in agreement. Coded transcripts were analyzed in Dedoose v9 [31].

**Fig. 1:**
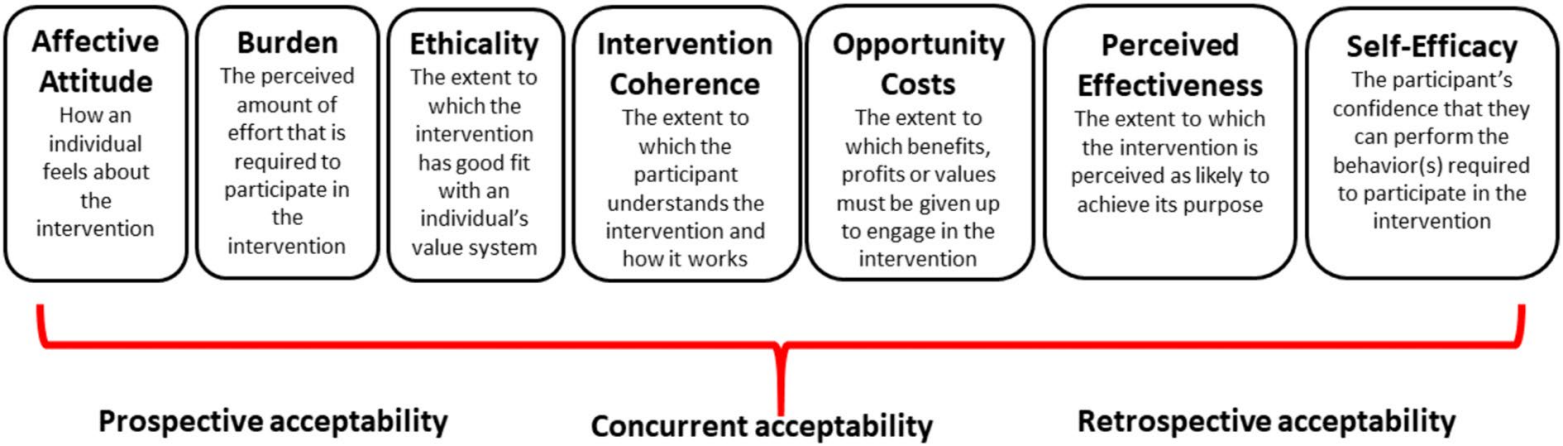
The Theoretical Framework of Acceptability (TFA), multi-construct framework for assessing prospective (i.e., anticipated) and retrospective (i.e., experienced) intervention/treatment acceptability [35]

This manuscript summarizes thematic findings from one code: injectable buprenorphine. Analysts [TS, RR, PF] employed final inductive and deductive strategies in reviewing the injectable buprenorphine code report. Data were reviewed independently by each analyst and emergent themes were derived using a data-driven thematic coding scheme in keeping with modified grounded theory [32, 33], and employing theory matching as described by Goldkuhl and Cronholm [34]. Colloquialisms and utterances were removed from quotes to improve readability. To aid the organization and interpretation of results, the analysts compared their summaries of emergent themes and utilized the *Theoretical Framework of Acceptability* (TFA) to provide a robust multi-construct framework to assess XR-Bup acceptability [35]. The TFA framework is comprised of seven constructs: affective attitude (feelings about an intervention), burden (effort required to participate), perceived effectiveness (perception that the intended purpose is achieved), ethicality (fit with an individual’s values), intervention coherence (understanding of the intervention), opportunity costs (forgone alternate potential benefits), and self-efficacy (confidence in one’s ability to participate). In addition, the multi-construct TFA framework allows for assessment of prospective (i.e., anticipated) and retrospective (i.e., experienced) intervention/treatment acceptability [35] among participants who have already received XR-Bup and those who have contemplated receiving such treatment. [Fig. 1].

## Results

### Participant characteristics

The 38 participants who completed in-depth interviews had a mean age of 41.5 (SD: 9.3) years, were 86% male, 84% White, 24% Hispanic, and 71% had completed a high school diploma or less. Nearly all participants (95%) reported taking MOUD at the time of the interview and, while 56% reported currently taking buprenorphine, and 11% reported currently taking XR-Bup (Table 1).

**Table 1:** Characteristics of participants who received medications for opioid use disorder while incarcerated in Massachusetts jails, 2022 (n = 38)

Characteristic	Count (%)
Age, mean (SD)	41.5 (9.3) Missing = 1
Female, n (%)	4 (14.3)
Race, n (%)	
White	32 (84.2)
Black or African American	3 (7.9)
Native Hawaiian or Other Pacific Islander	1 (2.6)
More than one race	2 (5.3)
Hispanic/Latino ethnicity, n (%)	9 (23.7)
Education, n (%)	
No high school diploma	8 (21.1)
High school diploma or equivalent	19 (50.0)
Some college, but no degree	9 (23.7)
Associate's degree	2 (5.3)
Not currently taking MOUD, n (%)	2 (5.3)
Currently taking MOUD, n (%)	36 (94.7)
Buprenorphine (e.g., Suboxone, Subutex)	20 (55.6)
Methadone	11 (30.6)
XR-Bup (Sublocade)	4 (11.1)
XR-Naltrexone (Vivitrol)	1 (2.8)
County of incarceration, n (%)	
A	1 (2.6)
B	6 (13.2)
C	4 (10.5)
D	8 (21.1)
E	3 (7.9)
F	6 (15.8)
G	3 (7.9)
H	7 (18.4)

**Fig. 2:**
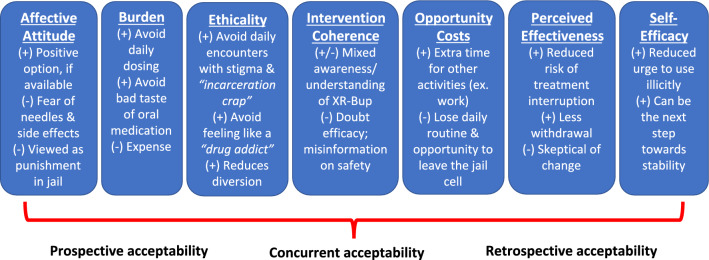
The Theoretical Framework of Acceptability of healthcare interventions, highlighting salient findings related to the acceptability of extended-release buprenorphine (XR-Bup) among people who received medication for opioid use disorder while incarcerated in Massachusetts jails

Salient themes for all seven constructs of the TFA emerged from the data, highlighting both positive and negative perspectives of and experiences with XR-Bup within the constructs (Fig. 2). Some of our participants assessed XR-Bup prospectively, noting what they anticipated based on what they had learned from others, while some participants described their actual XR-Bup experiences retrospectively.

### Affective attitudes: feelings about the intervention

Recently released participants reported split perspectives about XR-Bup as a treatment intervention, with approximately half viewing XR-Bup in a favorable light, while about half viewed it unfavorably. Among the positive affective attitudes, participants noted that XR-Bup was less habit forming:

I've heard from many people that it's the better way to go because then you have no habit forming and taking something every day and looking for it. It's just in your system. You don't need to do anything. Just give you a shot, and there you go and good for a month [Participant #: 403, SLB, continued MOUD in the community].

Further, participants indicated that they did not perceive a substantial difference between taking XR-BUP and SLB, noting that *“*…*it’s [XR-Bup] still the same medication, in jail it doesn't really matter…” [106, SLB, continued].*

Participants shared negative affective attitudes regarding needles, side effects, pain, and a waning effect near the end of the month. In considering injections of XR-BUP, one participant noted “*They were offering the shot…but I didn't go that route…I didn't want—I don't like needles in the first place, I never used them. I was addicted to pills” [203, SLB, continued].* Another noted that “…*it hurt, and I don’t know I just didn't like it,” [401, SLB, continued].* In reflecting on waning efficacy of the XR-Bup injections, one participant indicated that:

"…[XR-Bup] wasn’t enough. I had been fighting with them to try to up my dose, because it was at the end of my shot, and so they were trying to – they would not up my dose, they would not give me anymore...” [602, SLB, continued].

Several participants also indicated XR-Bup was viewed as a punishment in jail in that, *“…if you get in trouble, they kind of force you to do the Sublocade (XR-Bup),” [602, SLB, continued].* Typical ‘trouble’ was connected to getting caught trying to divert SLB or methadone. At some jails, XR-Bup was offered as the last treatment option for people with multiple diversion attempts. And yet, participants noted that *“…if that [XR-Bup] was the only option, then yes. But if it wasn't the only option, then I would just stick with the Subutex* [SLB without naloxone]” *[601, SLB, continued].*

…people cheeking their medication to sell it, you know what I mean, and then they get caught. They give you one chance, and you go to the hole [solitary confinement] and come back, but you'll still be on your medication. But they would drop it in half, like if you were on 16 and you got caught, they drop it to eight milligrams. And then if you got caught again, they would shut you off and give you the Sublocade shot [601, SLB, continued].

### Burden: effort required to participate

Participants appreciated the lower burden associated with XR-Bup, by avoiding daily dosing with SLB, indicating that “*A lot of people would take the shot because they figured they didn’t gotta get up every morning,” [301, SLB, continued].* Another participant talked about the inconvenience of attending daily dosing, and the demeaning experience surrounding SLB administration:

If you want to take suboxone, they may get shipped out 10 minutes, hands on the table and wait that 10-minutes until it’s totally dissolved. And then, they take Q-tip around your mouth and they make sure there isn’t anything in there…I don’t want to be put in that situation [505, XR-Bup, continued].

Participants liked not having the bad taste of oral medication: *“I’m not a huge [fan] of Suboxone, it’s disgusting. The taste is just unbelievable I'd say. It's something you don't get used to [305, SLB, discontinued].”* Others explained, *“…holding Suboxone in your mouth was frigging nasty…[203, SLB, continued]”* and that *“the taste of it makes me feel nauseous [202, SLB, continued].”*

An additional burden of XR-Bup was the cost. In the following interviewer-participant exchange, the participant identified the expense of the XR-Bup treatment as a barrier to continued injections:

Interviewer: You mentioned before that before you got there [jail], you were receiving the injectable, Sublocade. And you talked a little bit about like your experience and you said that you would have preferred that one, right? Like, if that you could continue it—

Participant: Oh yeah, absolutely yeah, but it's like super—it's like $1400, $1500 a month so they didn't, you know— obviously, they didn't offer it, you know, at either jail… [502, XR-Bup, continued].

### Ethicality: fit with an individual’s values

In pondering the extent to which the XR-Bup treatment was a good fit with an individual’s value system, participants indicated interest in avoiding daily encounters with externalized and internalized stigma and “incarceration crap” and being made to feel like a “drug addict”:

Just to avoid the daily crap of going down and you know searching your mouth and you know it's just, it's just this is more incarceration crap…So, just to avoid that everyday monotony of going and getting in all that [305, SLB, discontinued].

Another participant described the internalized stigma that she confronted, a *“…reminder that you're a loser every day…”* and how *“…XR-Bup could take it away, eliminating if it were available in jail, I 100% would take it [502, XR-Bup, continued].”*

Diversion reduction was also noted as a relevant benefit of XR-Bup among participants, diminishing frictional relationships and strong-arming among incarcerated individuals, and better fitting with the participant’s values related to diminished tensions, as noted by the following participant:

Maybe that [XR-Bup] would be a better option as far as people incarcerated to get on that because the only way to cheek that or give it out you got to cut yourself open and take it out. I don't really see inmates going to that extreme to do that...getting involved in buying suboxone off inmates and using them to get high not only does it cause problems for yourself, but it can cause a lot of negative behaviors between inmates. Fighting, arguing, just a lot of negativity that you probably don't want to deal with [501, XR-NTX, continued].

### Intervention coherence: understanding of the intervention

The extent to which the participants understand how the XR-Bup treatment works also plays a key role in their willingness to consider this treatment modality. We identified mixed awareness and understanding of XR-Bup among participants who noted that “*I don't know anything about it [506, SLB, continued],”* and others who asserted that *“For now, I'll stick with my oral use of Suboxone and I'll give it [XR-Bup] a thought, just trying to find out more information of this as time goes on and I might…consider it,”* [705*, SLB, continued*]. Yet others, who had a better understanding of XR-Bup treatment implications, noted that *“…it's long-acting, it's once a month, you wouldn't have to go there every single day. It's a time-release, I think it works probably better with your body chemistry [701, XR-Bup, continued].”*

Interviewees also shared some doubts regarding the perceived efficacy of the XR-Bup treatment, as well as safety concerns, and a perceived lack of effective dosing, with some participants indicating concerns about the long-term dose as they *“…don't like the idea of having something in you. And then, if God forbid, people had a bad reaction to it, it's already injected inside and it can’t be reversed [702, methadone, continued]”:*

I've heard a whole—a lot of horror stories about that [XR-Bup] because it's so much buprenorphine, that I don't know if the distribution of it, they've mastered like you know, because the stomach, I think it gets hot and it gets released faster than it should sometime—I don't know. I've heard horror stories about it [103, SLB, continued].

It [XR-Bup] didn’t really do much to me, I mean, it didn't make me sick, but I didn't feel like it gave me like any, it didn't take away like cravings or anything like that…Sublocade didn't really do anything, it felt like just something was going into my body, but for no reason [602, SLB, continued].

### Opportunity costs: forgone alternate potential benefits

Opportunity costs among participants, alluding to the extent to which benefits or values must be given up (or can be gained) to engage in XR-BUP treatment, revolved largely around time and routines. Participants noted that they need to devote less time to treatment on a daily basis, which could free up time for other activities, such as work:

I wanted the shot for a lot of reasons…It's a line waiting over here to get my medication. Takes up legitimately over a week, hours and hours of your time [505, XR-Bup, continued].

It [Suboxone] is more time consuming, and just taking it once a month, I could be working in the kitchen and then, to leave the kitchen every morning just to go get my Suboxone, it's kind of, a hassle, because it interferes with the job duty that you have for the day [705, SLB, continued].

On the other hand, some participants indicated concerns about losing a daily routine and the opportunity to leave their housing unit:

I like the habit, if you will, of having to take it every day. It keeps me more in my mind, you know, why I'm taking it and stuff versus, if it was a shot, I’d forget about it in a week and just go and start drinking or doing coke or something else, you know? I stick, I just, doing a daily thing. It’s a personal preference [603, SLB, continued].

I'd rather just get it every day than do it all at once and you got nothing to look forward to and hell, you know, what I mean? And so just that fact that you have a scheduled time to be out of your cell…[103, SLB, continued].

### Perceived effectiveness: perception that the intended purpose is achieved

Released participants perceived the effectiveness of XR-Bup in achieving its purpose (i.e., treatment for OUD) with some ambivalence. Some participants valued the reduced risk of treatment interruption that they perceived with XR-Bup, noting that *“Some people prefer that because they don't have to worry about missing a dose…” [102, methadone, discontinued]* and this was noted as especially important when experiencing major life and family changes:

I’m having, you know, I’m having a grandchild, you know, my first grandchild and it's, uh, it's very important for me…to be…sober and, and do I think that I could do it without the, the Sublocade injection? Yeah, maybe I could do it without it. But I'm not willing to take that risk…not yet. Maybe…down the road…[502, XR-Bup, continued].

Participants were optimistic about experiencing fewer issues with opioid withdrawal symptoms, mentioning that “*With the sublocade, it just comes out of your body and you’re with no withdrawal, as in you’re going through withdrawal with the suboxone* [801, XR-Bup, continued].”

Several participants were also skeptical about changes in treatment options and modalities, and they were not interested in changing their MOUD.

I personally would not [take sublocade]...I’m on methadone right now and I’ve been on a stable dose for quite some time and I’m doing very well on the medication that I’m on [304, methadone, continued].

### Self-efficacy: confidence in one’s ability to participate

Participant’s confidence that they can carry out the requirements to effectively take XR-BUP, or their self-efficacy required to participate in this treatment intervention, was exemplified by two major themes, noting interest in the reduced urge to use opioids illicitly, as well as beliefs that XR-Bup treatment could be the next step in their recovery and stability. One participant, contemplating XR-Bup treatment, noted positive feedback from a peer, appearing to boost confidence that the treatment could work them:

I heard that that also is a better thing because when they give you that shot, it comes into

your body so slowly that when you decide to get off it, you don't even get withdrawals or

anything because it just slowly goes into your body and it slowly goes out. So, I've heard great

things about that [403, SLB, continued].

## Discussion

Through qualitative interviews with people who were recently released from Massachusetts jails (n = 38) where they had received MOUD treatment, we identified a wide spectrum of positive and negative perspectives regarding XR-Bup as an OUD treatment option. We identified salient themes at all levels of the Theoretical Framework of Acceptability. From a positive perspective, participants shared favorable attitudes regarding avoidance of the bitter taste of SLB and daily dosing (burden), which had the added benefit of avoiding stigmatizing and demeaning “incarceration crap” with peers and jail staff, as well as daily reminders about being a “drug addict” (ethicality). Participants also favored less time devoted to daily treatment regimens and the extra time made available through monthly treatment for other activities, such as work (opportunity costs), and reduced risk of treatment interruption and withdrawal symptoms (perceived effectiveness), as well as positive steps towards treatment stability (self-efficacy) through XR-Bup treatment. On the negative side, several participants associated XR-Bup treatment with painful needles and punishment in the jail setting, serving as the last treatment option following attempts to divert SLB or methadone treatments (affective attitude), and being costly, rendering the treatment less accessible in jails (burden). They also noted concerns about a waning treatment effect near the end of the month and inadequate information regarding treatment safety and efficacy (intervention coherence), as well as lost daily opportunities to leave their cells (opportunity costs).

Our findings highlighted XR-Bup treatment ambivalence among patients across eight Massachusetts jails that were consistent, in several ways, with those highlighted by Cheng et al. in a New York City jail [24]. Cheng and colleagues identified salient themes across systems, medication, and patient-level factors within the context of an RCT in one jail, while we identified salient themes across all levels of the TFA within post-release community settings tied to eight jails. At the systems-level, Cheng found that key items tied to initiation of XR-Bup in jail included avoidance of perceived stigmatization and privacy concerns related to daily SLB administration, as well as diminished concerns with diversion, themes that were concordant with our ethicality construct, i.e., the perceived extent to which the intervention fits with one’s values. Interestingly, Cheng found that peer networks within jail favorably influenced participant adoption of XR-Bup treatment, while our participants pointed to peer pressures related to diversion and stigmatizing views. Diversion and coercion among MOUD patients within northeastern US jails have been reported previously [18, 21], and XR-Bup has been viewed as an effective option that can address administrative concerns about diversion [21]. Participant concerns about XR-Bup injection-related pain, as well as other side effects tied to discomfort are consistent with prior studies [21, 22, 24, 36]. And the perceived effectiveness of XR-Bup among our participants was consistent with the RCT conducted in New York City, which found that patients who received XR-Bup had fewer jail medical visits compared with patients who received daily SLB, and a two-fold higher 8-week retention rate in community buprenorphine treatment post-release when comparing patients in the XR-Bup group (69.2%) to the SLB group (34.6%) [22]. Similarly, a Rhode Island cohort study that found that 61% of patients released on XR-Bup engaged in MOUD post-release, and 30% continued with XR-Bup treatment [21].

Among our most novel findings were participant perceptions related to XR-Bup treatment as a punishment in the jail setting, serving as the MOUD treatment of last resort following attempts to divert SLB or methadone treatments. While prior research has reported no concerns with XR-Bup diversion in carceral settings [21], receiving a treatment as a “punishment” may contribute to mixed interests and motivations for XR-Bup continuity post-release from jail. Given that many jails in Massachusetts [27], in other US states [15, 37], and globally [12, 13, 38] are currently offering MOUD for OUD treatment, and some have begun to offer and assess the effectiveness of XR-Bup in carceral settings [21, 22, 36], a nuanced approach to providing XR-Bup appears merited. Such an approach could emphasize that the expense of XR-Bup limits its use to patients who have “ineffective experiences” with other treatments rather than those who are punished for “messing up”.

Indeed, participants reported concerns about the costs of XR-Bup, which appeared to hinder access to this treatment option in jails. To date, two studies have conducted costing analyses to assess comparative costs of SLB to XR-Bup, one within correctional facilities in Australia [20] where Buvidal® was available for weekly XR-Bup treatment, and one in the US that assessed costs of monthly XR-Bup (Sublocade®) treatment outside of correctional settings [39]. While the per dose cost of XR-Bup is higher than that for SLB, cost analyses in Australia indicated that XR-Bup (Buvidal) is more affordable in carceral settings than other MOUD formulations over the course of treatment [20]. In the US, and outside of correctional settings, Flam-Ross found that “at current medication cost and retention rates, XR-Bup was not associated with efficient allocation of limited resources when SLB was available [39].” It is important to note that a new weekly formulation of XR-Bup (Brixadi) is now available in the US and its cost is comparable to that of Sublocade, but it can be administered weekly or monthly, while Sublocade can only be administered monthly. Competitive pricing across products could lead to decreases in cost, which may make them more affordable and available within corrections settings. Another concern was the waning effectiveness of Sublocade near the end of the month. Prior reports suggest that up to 55% of patients on XR-Bup require supplementation with SLB, which may reduce some of the benefits of XR-Bup [40].

Despite the strengths of our study, our findings should be considered in light of a few limitations. While our sample size is relatively large (n = 38) and from a range of jails across Massachusetts, perspectives may differ from jails in other jurisdictions, potentially limiting transferability of findings. Participants’ time since release varied considerably, with some recently released and some released up to three years prior, and it is possible that participants who had been incarcerated in the distant past may have had less accurate recall of their experiences with MOUD and XR-BUP in jail. In addition, we present findings from participants who reflect on their experience within and outside of jails. This may contribute to a conflation of anticipated and actual use of XR-Bup in and outside of the jail setting, which may lead to conclusions that are less transferable to all settings. Finally, our sample included a relatively small number and percentage of female participants. This is largely because most of the participating jails (6 of 8) only housed incarcerated men. Future research is needed to better understand differences in XR-Bup perspectives and experiences among incarcerated women.

## Conclusion

People who received MOUD in Massachusetts jails had mixed views and experiences with XR-Bup. XR-Bup was well-received by some, but not all, people who had received MOUD in jail. Understanding these perceptions can inform protocols in jails that are considering implementation of XR-Bup. Our novel finding that participants perceived XR-Bup treatment as a punishment in the jail setting, serving as the MOUD treatment of last resort following attempts to divert SLB or methadone treatments, merits consideration as jails seek to improve MOUD treatment in ways that can enhance rather than detract from patient-centered treatment opportunities. Most prior studies have focused on safety, tolerability, side effects, and the effectiveness of the treatment, inside and outside carceral settings. Our study adds to the sparse literature on patient perspectives, which can inform development and implementation of XR-Bup treatment in jails, and has the potential to reduce overdose risks post-release.

## Supplementary Information

Additional file 1.

## Data Availability

Not applicable.
