# Supplementary material for: Perceptions of extended-release buprenorphine among people who received medication for opioid use disorder in jail: a qualitative study
Source: Addict Sci Clin Pract. 2024 Sep 12;19:68. doi: 10.1186/s13722-024-00486-2 (PMC11395844; doi:10.1186/s13722-024-00486-2)
Supplement: Supplementary file 1 — Additional file 1. [file 13722_2024_486_MOESM1_ESM.docx]

Appendix. COREQ (COnsolidated criteria for REporting Qualitative research) Checklist

| **Topic** | **Item No.** | **Guide Questions/Description** | **Page No.** | **Details** |
| --- | --- | --- | --- | --- |
| **Domain 1: Research team and reflexivity** | | | | |
| *Personal characteristics* | | | | |
| Interviewer/facilitator | 1 | Which author/s conducted the interview or focus group? | n/a | TS, RR (as well as several others who were not co-authors) |
| Credential | 2 | What were the researcher’s credentials? E.g., PhD, MD | 8 | PhD, MD, MPH, MS |
| Occupation | 3 | What was their occupation at the time of the study? | 8 | Social worker, anthropologist, clinical psychologist, public health PhD candidate, and masters-level staff members |
| Gender | 4 | Was the researcher male or female? | 8 | Females and males |
| Experience and training | 5 | What experience or training did the researcher have? | 8 | Prior experience conducting qualitative research interviews and years of substance use-focused research. |
| *Relationship with participants* | | | | |
| Relationship established | 6 | Was a relationship established prior to study commencement? | n/a | Interviewers introduced themselves as research team members to study participants. |
| Participant knowledge of the interviewer | 7 | What did the participants know about the researcher? e.g., personal goals, reasons for doing the research | n/a | Participants reviewed a consent form which included information about the study background and aims. |
| Interviewer characteristics | 8 | What characteristics were reported about the interviewer/facilitator? e.g., Bias, assumptions, reasons and interests in the research topic | n/a | The education levels, sex, and disciplines, of the interviewers is reported in the manuscript. |
| **Domain 2: Study design** | | | | |
| *Theoretical framework* | | | | |
| Methodological orientation and Theory | 9 | What methodological orientation was stated to underpin the study? e.g., grounded theory, discourse analysis, ethnography, phenomenology, content analysis | 8 | Emergent themes were derived using a data-driven thematic coding scheme iteratively developed by the analytical team in keeping with grounded theory |
| *Participant selection* | | | | |
| Sampling | 10 | How were participants selected? e.g., purposive, convenience, consecutive, snowball | 7 | Recruitment was conducted via flyers and word-of-mouth. |
| Method of approach | 11 | How were participants approached? e.g. face-to-face, telephone, mail, email | 7 | Flyers were posted or distributed in jail release packets and community locations where previously incarcerated individuals may gather (e.g., community opioid treatment programs (OTP), transitional housing). |
| Sample size | 12 | How many participants were in the study? | 7 | 38 |
| Non-participation | 13 | How many people refused to participate or dropped out? Reasons? | n/a | 63 people contacted the research team; 51 were eligible; 13 were lost to follow-up. |
| *Setting* | | | | |
| Setting of data collection | 14 | Where was the data collected? e.g., home, clinic, workplace | 8 | Phone |
| Presence of nonparticipants | 15 | Was anyone else present besides the participants and researchers? | n/a | No. |
| Description of sample | 16 | What are the important characteristics of the sample? e.g., demographic data, date | 9 | See table 1. |
| *Data collection* | | | | |
| Interview guide | 17 | Were questions, prompts, guides provided by the authors? Was it pilot tested? | n/a | Questions were developed by the authors. Pilot testing was not conducted, however senior most team members conducted the initial interviews. |
| Repeat interviews | 18 | Were repeat interviews carried out? If yes, how many? | n/a | Repeat interviews were not carried out. |
| Audio/visual recording | 19 | Did the research use audio or visual recording to collect the data? | 8 | Audio recordings were used. |
| Field notes | 20 | Were field notes made during and/or after the interview or focus group? | n/a | Notes were taken by interviewers. |
| Duration | 21 | What was the duration of the interviews or focus group? | 8 | Interviews lasted 30-60 minutes. |
| Data saturation | 22 | Was data saturation discussed? | n/a | In lieu of discussion on data saturation, we provide ample details on our sample characteristics and highlight salient findings. |
| Transcripts returned | 23 | Were transcripts returned to participants for comment and/or correction? | n/a | The transcripts were not returned to participants for comment and/or correction |
| **Domain 3: analysis and findings** | | | | |
| *Data analysis* |  |  |  |  |
| Number of data coders | 24 | How many data coders coded the data? | 8 | 4 coders (2 dyads) |
| Description of the coding tree | 25 | Did authors provide a description of the coding tree? | 8 | Codes were refined using open coding and constant comparative methods, resulting in a codebook with 23 parent codes and 32 child codes. |
| Derivation of themes | 26 | Were themes identified in advance or derived from the data? | 8 | Emergent themes were derived using a data-driven thematic coding scheme iteratively developed by the analytical team in keeping with grounded theory |
| Software | 27 | What software, if applicable, was used to manage the data? | 8 | Dedoose v9 (Los Angeles, CA) |
| Participant checking | 28 | Did participants provide feedback on the findings? | n/a | No. |
| *Reporting* | | | | |
| Quotations presented | 29 | Were participant quotations presented to illustrate the themes/findings? Was each quotation identified? e.g., participant number | n/a | Yes. |
| Data and findings consistent | 30 | Was there consistency between the data presented and the findings? | n/a | Yes |
| Clarity of major themes | 31 | Were major themes clearly presented in the findings? | n/a | Yes |
| Clarity of minor themes | 32 | Is there a description of diverse cases or discussion of minor themes? | n/a | Yes |

Developed from: Tong A, Sainsbury P, Craig J. Consolidated criteria for reporting qualitative research (COREQ): a 32-item checklist for interviews and focus groups. International Journal for Quality in Health Care. 2007. Volume 19, Number 6: pp. 349 – 357
